# Measurement, source decomposition, and formation mechanism of differences in the development of China’s health services

**DOI:** 10.3389/fpubh.2025.1495077

**Published:** 2025-03-26

**Authors:** Fulei Jin, Xigang Zhang, Yiming Zhao

**Affiliations:** ^1^School of Labor Relations, Shandong Management University, Jinan, China; ^2^School of Economics, Shandong University of Finance and Economics, Jinan, China

**Keywords:** health services, spatial differences, structural differences, formation mechanism, health equity

## Abstract

In China, the issue of unbalanced development is rather prominent. This paper conducts research on the differences in China’s health service development, aiming to provide references for promoting health equity through the equalization of health services. A series of quantitative methods are applied in this study, including the two-stage entropy method, spatial kernel density estimation, Theil index, variance decomposition method, and quadratic assignment procedure (QAP). Based on publicly-available macro data such as the China Health Statistics Yearbook, the two-stage entropy method is employed to comprehensively measure the health service levels of 31 provinces in China from 2005 to 2019. Then, spatial density estimation is used to examine the distribution characteristics of China’s health services. The Theil index and variance decomposition methods are utilized to explore the sources and magnitudes of the differences in the development of health services from both spatial and structural perspectives. Additionally, the QAP is used to examine the driving factors of the differences in the development of health services across the country as well as in the eastern, central, and western regions. The research findings are as follows: (1) The development levels of health services in the whole country and the three major regions show an upward trend. However, there are significant imbalances among the three regions, with the development level of health services in the eastern region consistently higher than that in the other two regions. (2) Concerning spatial differences, the intra-regional differences are the main source of the overall differences. Among them, the internal differences in the eastern region are the largest, followed by those in the western region. (3) In terms of structural differences, the differences in curative ability are the main source of the overall differences. The contributions of differences in personnel, service utilization, and primary services to the overall differences decrease in sequence. (4) Facilities in the eastern region have the greatest impact on overall differences, while differences in personnel in the central and western regions have the greatest impact on overall differences.

## 1 Introduction

Health is one of the basic rights of human beings, and people’s health serves as the foundation for social development. With the intensification of population aging, people’s demand for health services has been continuously increasing. Taking 2023 as an example, the total national health expenditure reached 9.06 trillion yuan, accounting for 7.2% of GDP. However, although the scale of China’s health services has witnessed rapid growth, there are obvious problems of unbalanced development. Taking the number of physicians as an example, in 2023, the number of practicing (assistant) physicians per 10,000 population in Beijing was 61.2, while in Yunnan it was 31.4, a difference of 1.95 times. As an important part of basic public services, the balanced development of health services is related to the health and well-being of the general public. It has a significant impact on safeguarding citizens’ right to health, maintaining social fairness and justice, and promoting common prosperity. At present, the principal contradiction in Chinese society has evolved into the one between the people’s ever - growing needs for a better life and unbalanced and inadequate development. The unbalanced development of health services is one of the manifestations of this principal contradiction. In order to achieve the vision of “health for all,” in 2016, the State Council issued the “Healthy China 2030” Plan Outline, emphasizing the promotion of equal access to basic public health services. Against this backdrop, this paper focuses on the following core issues: (1) Construct a comprehensive indicator system to comprehensively measure the development level of China’s health services, and analyze its evolution trends and distribution characteristics. (2) Decompose the differences in the development of China’s health services from spatial and structural perspectives. (3) Explore the formation mechanisms behind the differences in the development of China’s health services.

Quantitatively measuring the development level of health services is the basis for this research. By reviewing relevant literature, it can be found that many scholars often use methods such as the Data Envelopment Analysis (DEA) to evaluate the development of health services from the perspective of efficiency. In terms of inputs, indicators from three aspects of funds, human-resources, and health resources are usually selected (1), including the number of health personnel, the assets of health institutions, and the total health expenditure. In terms of outputs, indicators such as the number of outpatient visits and the number of in-patients are usually chosen (2–5). This paper argues that although the improvement of efficiency is an important aspect of the development of health services, from the conclusions of existing research, a single efficiency indicator obviously cannot meet the research needs for health services. First, health is a basic human need. Health services are different from general production activities. They have not only economic attributes but also special public attributes, and it is not appropriate to evaluate them with simple efficiency thinking. Second, there are deficiencies in some calculation processes, resulting in over-estimated efficiency evaluations. Take the research of Liu as an example (6). The measurement results of this study show that provinces with a large population base and some western provinces have relatively high health service efficiency. For example, Shandong and Henan rank the top two, and Tibet ranks fifth, while Beijing, which is rich in health resources, only ranks 30th. This is obviously inconsistent with the actual situation. There are two reasons for this phenomenon: First, when there is a large population but insufficient investment in health resources, a large number of health-service demanders cannot access services, causing the entire health system to operate overloaded. In this case, the calculated efficiency value will inevitably be high, but this high efficiency is at the expense of patient treatment. Second, areas with insufficient health resources often cannot provide adequate care for patients, while patients in areas with well-developed health services can receive long-term and adequate care. This leads to the statistical phenomenon of high efficiency in backward areas, but this high efficiency is due to the insufficient health services received by patients. Therefore, when the total amount of health resources in China is still relatively scarce, using a single efficiency indicator cannot reasonably evaluate China’s health services.

Based on the above discussion, this paper constructs an evaluation index system for comprehensive evaluation to overcome the evaluation bias problems caused by a single efficiency indicator and thus more truthfully reflect the development status of China’s health services. Compared with the method of using a single-indicator evaluation, the evaluation results of comprehensive evaluation are obviously more comprehensive and closer to the objective reality (7). Some scholars have evaluated the development of health services by constructing multi - dimensional comprehensive evaluation index systems. The research involves tertiary first - class hospitals, public hospitals, general hospitals, traditional Chinese medicine hospitals, primary - care services, etc. (8–13). Although the above-mentioned comprehensive evaluations of health services have achieved certain results, there is still room for optimization and improvement. First, in terms of research perspectives, most studies focus on the national overall level, and there is a lack of medical service evaluation systems at the regional level, making it difficult to analyze the regional differences in the development of health services. Second, in terms of index construction, there is a lack of comprehensive and systematic measurements of the development level of health services from multiple dimensions, and the selection of weights is highly subjective, making it difficult to accurately reflect the basic characteristics of the development of health services in China.

Regarding the unbalanced development of health services, existing studies have shown that the problem of unbalanced spatial distribution of health service development exists in many countries around the world (14–16). Studies on China mainly start from the perspective of regional differences. By applying methods such as the Theil index, Gini coefficient, and Kernel density estimation, it has been found that there are spatial non - equilibrium phenomena in China’s health services in terms of fiscal expenditure, resource allocation, allocation efficiency, service efficiency, and high - quality resources. However, with the implementation and promotion of policies such as “regional balanced development” and “equalization of basic public services,” the problem of regional differences has been alleviated to a certain extent (17–21). There are also studies that use methods such as Moran’s I index and LISA to analyze the spatial correlation pattern, and it has been found that the overall distribution of health resources in China shows the characteristics of HH and LL clustering (22, 23). The above-mentioned studies show that the problem of unbalanced development of China’s health services has been confirmed and has attracted the attention of scholars, but there are still two aspects that need to be expanded. First, most of the existing literature decomposes and examines health services from the perspective of spatial differences, and there are few studies that combine the spatial and structural perspectives for investigation. Second, in terms of research methods, the existing literature mostly involves the measurement of differences in the development of health services, but few studies involve the driving factors of unbalanced development.

In terms of the choice of spatial scale, the two main spatial scales in existing research are cities and provinces. Although city-level research is more refined in scale, it is often restricted by data availability. Currently, there is a lack of indicators that can be used to compare city-level medical services across the country over a long-span period (22). The few existing indicators such as the number of medical and health institutions, the number of hospital beds, and the number of doctors are relatively basic and rough. Therefore, these types of indicators are more suitable for evaluating the basic level of health services rather than the comprehensive level (23, 24). Although some studies have used more comprehensive and systematic city-level data (25–27), such studies are limited to a small spatial area or a short time range, and it is impossible to grasp the overall spatio-temporal pattern of China’s health service development from an overall perspective. In comparison, taking the provincial level as the research scale, on the one hand, it has a rich data foundation, which improves the feasibility and accuracy of the research. On the other hand, it is more in line with China’s national conditions and the characteristics of the health service industry. China’s medical insurance implements local management, and there are still certain institutional constraints on cross-provincial off-site medical treatment, which greatly limits the accessibility of health services outside the province. However, the problem of off-site medical treatment within the province has long been solved, and health services have strong sharing within the province (28). The concentration of advanced medical resources and talents in large and medium-sized cities within the province is conducive to giving play to the agglomeration effect of medical services and improving the health level of residents (21, 29, 30). Therefore, taking the provincial level as the research scale is more practically significant.

Based on the above discussions, this paper conducts research on the level measurement of China’s health service development, spatial and structural differences, as well as the driving factors of development differences, aiming to promote the balanced development of China’s health services. In this study, an index evaluation system is constructed, and the two-stage entropy method is adopted to measure the development level of health services in 31 provinces of China. Subsequently, the Theil index and variance decomposition method are employed to explain the magnitude and sources of differences in the development of China’s health services from both spatial and structural perspectives. Additionally, the QAP is used to explore the formation mechanism of unbalanced development. Compared with existing research, the possible contributions of this paper include the following aspects: (1) Starting from the influencing factors of health services, a comprehensive evaluation system is constructed based on five dimensions: personnel, facilities, curative ability, primary services, and service utilization. Then, the two-stage entropy method is adopted for level measurement. The amount of information contained in an indicator is measured by its entropy value. Generally, the smaller the entropy value of a certain indicator, the more information it provides. Thus, the more important it is in the comprehensive evaluation, and the greater its weight. The two-stage entropy method uses information entropy to calculate and allocate weights at the dimension level and the indicator level, respectively. Therefore, it can overcome the drawbacks of traditional entropy methods and accurately and comprehensively evaluate the development level of health services in various provinces of China. (2) The Theil index is used to measure the spatial differences in the development of China’s health services, and it is decomposed into intra-regional differences and inter-regional differences, thereby revealing the spatial sources of differences in the development of the health service industry and providing decision - making references for promoting regional coordinated development. (3) The variance decomposition method is used to reveal the structural sources of differences in the development of China’s health services, so as to identify the key areas for promoting the balanced development of health services. (4) Each province can be regarded as an actor. Thus, the disparities in the development of health services among provinces embody a relationship between two actors. By using QAP regression analysis based on relational data, this study reveals the formation mechanism of the disparities in the development of health services from the perspective of social network relationships. This provides support for promoting the orderly and balanced development of health services in a way that suits local conditions.

## 2 Materials and methods

### 2.1 Data source and regional division

This study is based on health data from 31 provinces in China, and the sample period is delineated as 2005 to 2019 in order to avoid the disruption of health system shocks by the COVID-19 epidemic. The China Health and Wellness Statistical Yearbook and the official website of the Bureau of Statistics are the data sources for this paper. Since the development of health services in each region is continuous, a small number of missing values in the sample are processed using the interpolation method. This ensures the integrity of the measurement results without interfering with the measurement accuracy. It should be noted that due to data availability limitations, regions such as Hong Kong and Macau are not included in this study, which may lead to potential biases.

According to the 1986 “Seventh Five-Year Plan,” China is divided into three regions: East, Central and West. Specifically, the eastern region consists of 11 provinces: Beijing, Tianjin, Hebei, Shanghai, Jiangsu, Zhejiang, Fujian, Shandong, Guangdong, Hainan, and Liaoning; the central region consists of 8 provinces: Heilongjiang, Jilin, Shanxi, Anhui, Jiangxi, Henan, Hubei, and Hunan; and the western region consists of 12 provinces: Inner Mongolia, Guangxi, Chongqing, Sichuan, Guizhou, Yunnan, Tibet, Shaanxi, Gansu, Qinghai, Ningxia, and Xinjiang.

### 2.2 The two-stage nested entropy method

The two-stage entropy method is employed to measure the development level of health services, and the specific steps are as follows:

To ensure comparability between the indicators of different units, it is necessary to perform dimensionless processing on the original data. Equations 1, 2 represent the dimensionless processing methods for forward-oriented and backward-oriented indicators, respectively.


(1) $$p{'}_{\mathrm{itjk}}=\frac{x_{\mathrm{itjk}}-\min\left(x_{\mathrm{itjk}}\right)}{\max\left(x_{\mathrm{itjk}}\right)-\min\left(x_{\mathrm{itjk}}\right)}$$

(2) $$p{'}_{\mathrm{itjk}}=\frac{\max\left(x_{\mathrm{itjk}}\right)-x_{\mathrm{itjk}}}{\max\left(x_{\mathrm{itjk}}\right)-\min\left(x_{\mathrm{itjk}}\right)}$$

Where $x_{\mathrm{itjk}}$ is the original index value of the k−th index of the dimension j in province i at period t.

The construction formula of a normalized matrix $p_{\mathrm{itjk}}$ using Equation 3:


(3) $$p_{\mathrm{itjk}}=\frac{p{'}_{\mathrm{itjk}}}{\sum_{t=1}^{T}\sum_{i=1}^{N}p{'}_{\mathrm{itjk}}}$$

Calculate the entropy of the k−th indicator of the dimension j using Equation 4:


(4) $$e_{jk}=-\frac{1}{\ln\left(TN\right)}\overset{T}{\underset{t=1}{\sum }}\overset{N}{\underset{i=1}{\sum }}p_{\mathrm{itjk}}\ln p_{\mathrm{itjk}}$$

Calculate the entropy of dimension j using Equations 5, 6:


(5) $$g_{itk}=\frac{\sum_{k=1}\frac{1-e_{jk}}{\sum_{j=1}^{J}e_{jk}}p_{\mathrm{itjk}}}{\sum_{t=1}^{T}\sum_{i=1}^{N}\sum_{k=1}^{K}\frac{1-e_{jk}}{\sum_{j}^{J}e_{jk}}p_{\mathrm{itjk}}}$$

(6) $$e_{j}=-\frac{1}{\ln\left(TN\right)}\overset{T}{\underset{t=1}{\sum }}\overset{N}{\underset{i=1}{\sum }}g_{itk}\ln g_{ikt}$$

Finally, the development level of health services is calculated using Equation 7:


(7) $$M_{\mathrm{it}}=\overset{J}{\underset{j=1}{\sum }}\frac{1-e_{j}}{\sum_{j=1}^{J}\left(1-e_{j}\right)}g_{itk}$$

### 2.3 Spatial kernel density estimation

The Kernel density estimation method is an important non-parametric estimation method. It can effectively reveal the dynamic evolution of the distribution of the development of China’s health services. The formula for traditional Kernel density estimation is:


(8) $$f\left(x\right)=\frac{1}{Nh}\overset{N}{\underset{i=1}{\sum }}K\left(\frac{X_{i}-x}{h}\right)$$

In Equation 8, $K\left(x\right)$ is the Gaussian Kernel function, and *h* is the bandwidth. Spatial Kernel density estimation incorporates temporal and spatial factors on the basis of traditional Kernel density estimation. Specifically, it uses a continuous density curve to describe the distribution pattern of the random variable *X* under spatio-temporal conditions, and the calculation formula is:


(9) $$g\left(y|x\right)=\frac{f\left(x,y\right)}{f\left(x\right)}$$

(10) $$f\left(x,y\right)=\frac{1}{Nh_{x}h_{y}}\overset{N}{\underset{i=1}{\sum }}K_{x}\left(\frac{X_{i}-x}{h_{x}}\right)K_{y}\left(\frac{Y_{i}-x}{h_{y}}\right)$$

### 2.4 Theil index

The Theil index, developed by Theil, is a method that employs the entropy concept in information theory to measure the disparity level. In this paper, the Theil index is applied to conduct spatial disparity analysis, which can reveal the sources of spatial disparities in the development of China’s health services. The relevant calculation formulas are shown in Equations 11–15:


(11) $$T=\frac{1}{n}\overset{n}{\underset{i=1}{\sum }}\frac{y_{i}}{y}\log\left(\frac{y_{i}}{y}\right)$$

(12) $$T_{k}=\frac{1}{n_{k}}\overset{n_{k}}{\underset{i=1}{\sum }}\left(\frac{y_{i}^{k}}{{\bar{y}}^{k}}\right)\log\left(\frac{y_{i}^{k}}{{\bar{y}}^{k}}\right)$$

(13) $$T_{w}=\overset{m}{\underset{k=1}{\sum }}\left(\frac{n_{k}}{n}\frac{{\bar{y}}^{k}}{\bar{y}}\right)T_{k}$$

(14) $$T_{b}=\overset{m}{\underset{k=1}{\sum }}\frac{n_{k}}{n}\left(\frac{{\bar{y}}^{k}}{\bar{y}}\right)\log\left(\frac{{\bar{y}}^{k}}{\bar{y}}\right)$$

(15) $$T=T_{w}+T_{b}$$

The Theil index ranges from 0 to 1. The larger the value, the greater the regional disparity. Equation 15 indicates that the overall Theil index is equal to the sum of the intra-regional Theil index and the inter-regional Theil index, reflecting the decomposable characteristic of the Theil index.

### 2.5 Variance decomposition method

The variance decomposition method is used in this paper to analyze the structural differences of health services. The development level (I) of health services can be decomposed into personnel index ($I_{1}$) facility index ($I_{2}$) curative ability index ($I_{3}$) primary services index ($I_{4}$) and service utilization index ($I_{5}$) to explore the main causes of the unbalanced development structure of health services. The calculation process is as shown in Equations 16, 17.


(16) $$\begin{array}{l} var\left(I\right)=cov\left(I,I_{1}+I_{2}+I_{3}+I_{4}+I_{5}\right)\\ =cov\left(I,I_{1}\right)+cov\left(I,I_{2}\right)+cov\left(I,I_{3}\right)+cov\left(I,I_{4}\right)+cov\left(I,I_{5}\right) \end{array}$$

(17) $$1=\frac{cov\left(I,I_{1}\right)}{var\left(I\right)}+\frac{cov\left(I,I_{2}\right)}{var\left(I\right)}+\frac{cov\left(I,I_{3}\right)}{var\left(I\right)}+\frac{cov\left(I,I_{4}\right)}{var\left(I\right)}+\frac{cov\left(I,I_{5}\right)}{var\left(I\right)}$$

Where $var\left(I\right)$ represents variance and $cov\left(I,I_{n}\right)$ represents covariance. Equation 11 is used to decompose the development differences of health services into sub-differences in five dimensions. Equation 12 is used to measure the contribution degree of each dimensional difference in the overall imbalance.

### 2.6 Quadratic assignment procedure (QAP)

The QAP is used to analyze the formation mechanisms of developmental differences in health services. Considering each province as an actor, the developmental differences between provinces can be regarded as a relationship between actors, and the values of level differences between all provinces can be formed into a matrix. In this way, the driving factors of the developmental differences in China’s health services can be explored. The relational data model is shown in Equation 18.


(18) $$Y=\beta_{0}+\beta_{1}X_{1}+\beta_{1}X_{2}+\beta_{1}X_{3}+\beta_{1}X_{4}+\beta_{1}X_{5}+U$$

Where Y is the explanatory variable, i.e., the matrix of differences in the level of development of health services, $\beta_{0}$ and $\beta_{1}$ are the parameters to be estimated, $X_{n}$ is the explanatory variable, and U is the residual term. Variables with observed values as relational data often have a serious problem of multicollinearity. For traditional statistical test methods, the variance and standard deviation of the parameter estimates increase, rendering the significance test of variables meaningless. However, the QAP does not require the assumption of independence among variables and can solve the problems of autocorrelation and multicollinearity in the econometric models of relational data. It should be noted that the QAP method is highly sensitive to outliers. Therefore, in this paper, the sample data was rigorously tested to eliminate the influence of outliers.

## 3 Measurement of the development level of health services in China

### 3.1 The evaluation index system

Based on the industry characteristics of health services, taking into account rationality and data availability, this paper selects 15 indicators from five dimensions of personnel, facilities, curative ability, primary services, and service utilization to construct an evaluation system for the development of health services. Regarding evaluation methods, the two-stage entropy method is used to calculate the entropy values of the indicator layer and the dimension layer to achieve objective weighting of indicators. The specific construction method of the evaluation system is shown in Table 1.

**Table 1:** The evaluation index system of the development level of health services.

Target layer	Dimension layer	Indicator layer		
		Indications	Unites	Attribute
The development level of health services	Personnel	Number of licensed or assistant doctors per thousand people	Individuals	+
		Number of registered nurses per thousand people	Individuals	+
		Number of health management personnel per thousand people	Individuals	+
	Facilities	Per capita assets of health institutions	Yuan	+
		Number of beds in health institutions per thousand people	Unit	+
		Number of hospitals per thousand people	Unit	+
	Curative ability	Maternal mortality rate	‰	−
		Perinatal mortality rate	‰	−
		Number of third-class hospitals per thousand people	Unit	+
	Primary services	Average number of staff in clinics in each village	Unit	+
		Number of primary medical institutions per thousand people	Unit	+
	Service utilization	Daily visits per doctor	Times	+
		Daily inpatients per doctor	Days	+
		The bed utilization ratio	%	+
		The average length of stay	Days	−

### 3.2 Measurement results and basic facts

**Figure 1:**
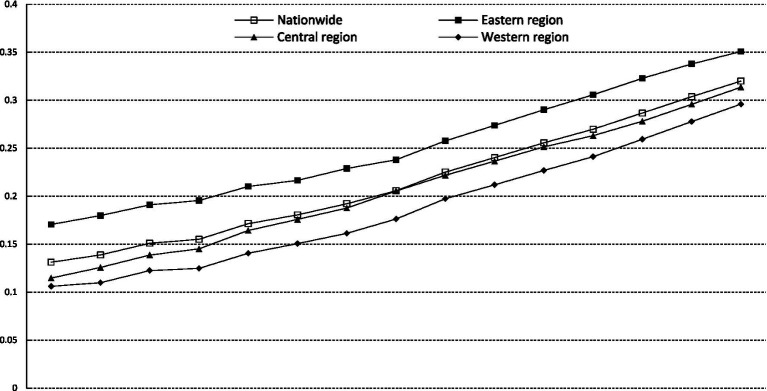
The evolution trend of the development level of health services.

Based on the above evaluation system, the two-stage entropy method is adopted to measure the development levels of health services in 31 provinces of China. Table 2 reports some of the measurement and ranking results. Then, this paper measures the average value of the development level of health services in the country and the three major regions from 2005 to 2019. Figure 1 reflects the trend of the evolution of the development level of health services. From the national level, the mean value of the development level of health services has increased year by year during the sample period, with an average annual increase of 6.58%. At the regional level, the mean value of the level of development of health services has also shown an increasing trend, with an average annual growth rate of 5.29% in the eastern region, 7.45% in the central region, and 7.60% in the western region, respectively. Based on the growth rates of the three major regions and the development levels of health services in these regions at the beginning of the sample period, the differences in health services among the three major regions show a shrinking trend. This indicates that the degree of equalization of health services is constantly increasing. However, there are obvious differences in the mean values of each region, with the eastern region always having the highest, followed by the central region. Overall, the development level of China’s health services has increased steadily during the sample period, but with significant regional imbalances.

**Table 2:** Measurement results and rankings of the development of China’s health services.

	2005		2007		2009		2011		2013		2015		2017		2019	
	Value	Rank	Value	Rank	Value	Rank	Value	Rank	Value	Rank	Value	Rank	Value	Rank	Value	Rank
Beijing	0.34	1	0.36	1	0.36	1	0.36	1	0.39	1	0.43	1	0.48	1	0.52	1
Tianjin	0.23	2	0.22	4	0.23	4	0.25	4	0.26	9	0.30	8	0.32	9	0.34	11
Hebei	0.12	14	0.14	15	0.15	20	0.17	19	0.19	21	0.21	22	0.26	21	0.29	21
Shanxi	0.11	19	0.15	12	0.20	7	0.21	11	0.24	12	0.26	14	0.30	13	0.32	15
Inner Mongolia	0.12	17	0.14	16	0.16	16	0.17	18	0.21	18	0.24	16	0.28	16	0.33	14
Liaoning	0.22	3	0.22	3	0.23	3	0.24	5	0.29	3	0.30	6	0.34	6	0.36	8
Jilin	0.15	8	0.17	9	0.18	12	0.19	14	0.22	15	0.26	15	0.28	15	0.33	13
Heilongjiang	0.16	7	0.18	7	0.20	8	0.23	8	0.28	4	0.31	4	0.35	5	0.38	5
Shanghai	0.21	4	0.23	2	0.25	2	0.26	2	0.28	6	0.30	7	0.32	10	0.34	10
Jiangsu	0.14	10	0.18	8	0.21	6	0.24	6	0.27	7	0.30	10	0.33	8	0.37	6
Zhejiang	0.13	12	0.17	10	0.19	9	0.24	7	0.28	5	0.33	2	0.35	4	0.36	7
Anhui	0.06	30	0.09	29	0.12	29	0.15	24	0.18	25	0.21	23	0.23	25	0.27	23
Fujian	0.09	28	0.09	28	0.17	13	0.17	21	0.18	24	0.20	25	0.21	30	0.24	30
Jiangxi	0.08	29	0.09	30	0.13	26	0.16	22	0.19	23	0.20	28	0.22	28	0.25	27
Shandong	0.12	15	0.14	14	0.16	17	0.19	15	0.24	13	0.30	11	0.36	2	0.42	2
Henan	0.11	21	0.12	21	0.14	22	0.17	17	0.22	16	0.23	18	0.26	20	0.30	19
Hubei	0.14	9	0.17	11	0.19	11	0.21	12	0.24	11	0.31	5	0.32	11	0.34	12
Hunan	0.10	23	0.13	19	0.15	19	0.17	20	0.20	19	0.24	17	0.26	17	0.31	17
Guangdong	0.17	6	0.21	5	0.22	5	0.25	3	0.29	2	0.32	3	0.36	3	0.38	4
Guangxi	0.12	13	0.14	18	0.15	18	0.18	16	0.21	17	0.22	20	0.24	23	0.27	24
Hainan	0.12	18	0.12	20	0.13	23	0.15	27	0.17	28	0.19	29	0.21	29	0.24	31
Chongqing	0.09	24	0.12	23	0.13	24	0.16	23	0.19	20	0.23	19	0.26	18	0.30	20
Sichuan	0.12	16	0.14	17	0.17	15	0.21	10	0.27	8	0.30	9	0.33	7	0.40	3
Guizhou	0.04	31	0.07	31	0.08	31	0.11	31	0.19	22	0.22	21	0.25	22	0.28	22
Yunnan	0.09	26	0.10	27	0.11	30	0.13	29	0.17	29	0.21	24	0.26	19	0.30	18
Tibet	0.11	20	0.11	25	0.12	28	0.13	30	0.14	31	0.18	31	0.21	31	0.25	28
Shaanxi	0.14	11	0.15	13	0.17	14	0.20	13	0.25	10	0.27	12	0.30	12	0.34	9
Gansu	0.09	27	0.10	26	0.13	27	0.13	28	0.16	30	0.20	26	0.23	26	0.26	25
Qinghai	0.09	25	0.11	24	0.13	25	0.15	25	0.18	26	0.20	27	0.24	24	0.26	26
Ningxia	0.11	22	0.12	22	0.14	21	0.15	26	0.17	27	0.19	30	0.23	27	0.25	29
Xinjiang	0.17	5	0.18	6	0.19	10	0.21	9	0.24	14	0.26	13	0.29	14	0.31	16

## 4 Decomposition of differences in the development of health services in China

In this section, the visualization analysis of ARCGIS software and the spatial Kernel density estimation method are utilized to unveil the distribution characteristics of the development of China’s health services. Subsequently, the Theil index method is applied to explore the sources of spatial differences in the development of China’s health services. Finally, the variance decomposition method is employed to examine the sources of structural differences in the development of China’s health services.

### 4.1 Distribution characteristics

**Figure 2:**
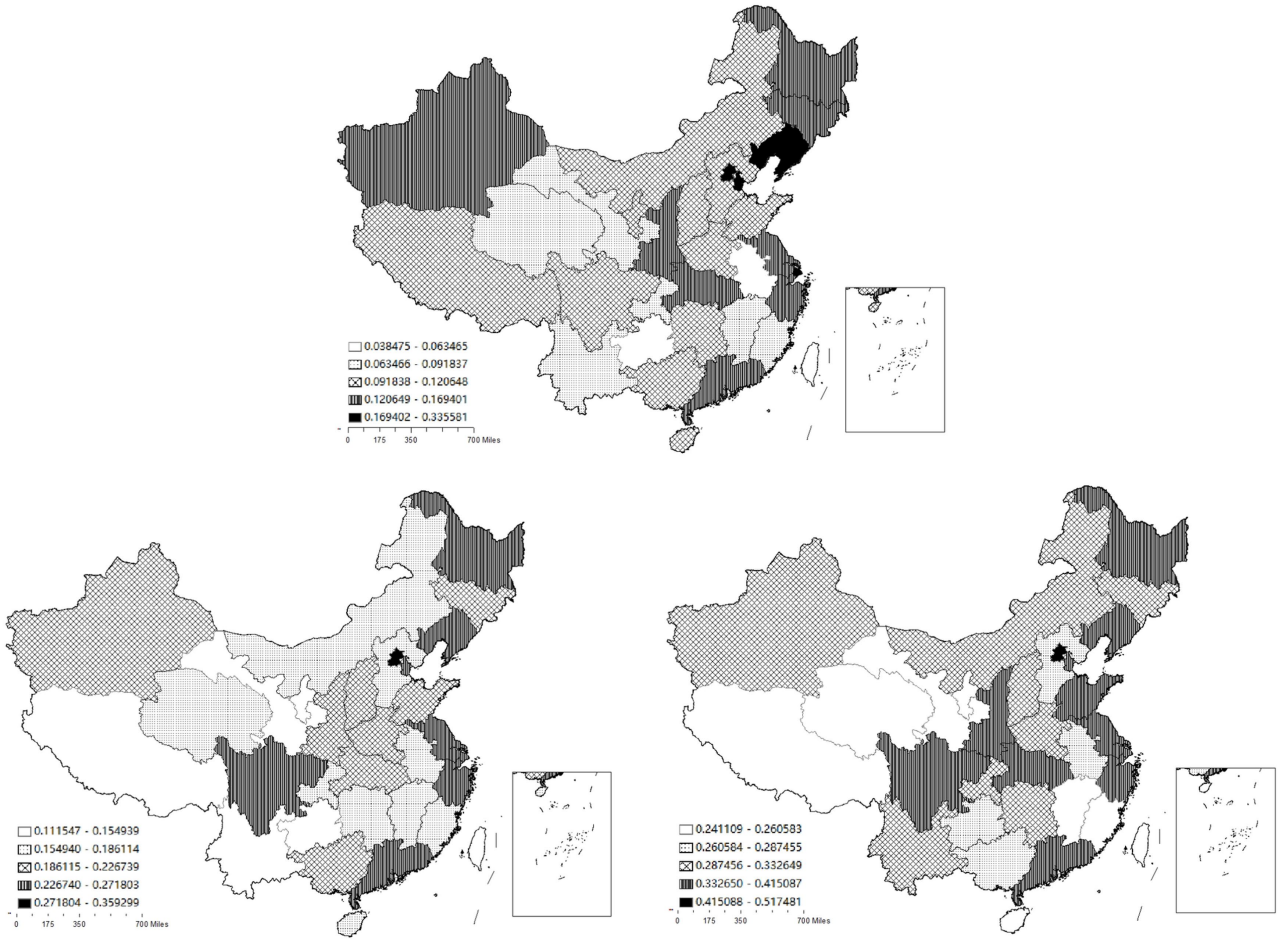
The spatial distribution of the development of China’s health services in 2005, 2012 and 2019.

This paper selects the cross-sectional data of various regions in China for 2005, 2012, and 2019, and adopts the natural breaks method to observe the spatial distribution differences in the development of health services. As can be seen from Figure 2, in 2005, the overall development level of China’s health services is relatively low. The highest value is 0.34 in Beijing, and the lowest is 0.04 in Guizhou. There are significant differences among regions. The high-value areas of health service development are mainly concentrated in regions such as Beijing, Tianjin, Shanghai, and Liaoning. As time progresses, in 2012 and 2019, the health service levels across the country generally increase and the centers become more dispersed. Taking 2019 as an example, apart from Beijing, Tianjin, Shanghai, and Liaoning, regions like Heilongjiang, Shandong, Jiangsu, Zhejiang, Shaanxi, Hubei, Sichuan, and Guangdong also have relatively high - level health services, presenting a scenario of “prospering in multiple locations.” The highest value is 0.52 in Beijing, and the lowest is 0.24 in Hainan. This indicates that over time, the overall level of China’s health services has been continuously improving, and simultaneously, there is a trend of decentralization in distribution.

To examine the spatial distribution characteristics of the development of China’s health services in more detail and present a three - dimensional display, this section further conducts research from two aspects: spatial statics and spatial dynamics based on the spatial Kernel density estimation method. Compared with the traditional Kernel density estimation, spatial Kernel density analysis has the advantage of simultaneously analyzing spatial and temporal information, and can reveal the spatial distribution pattern of the development of China’s health services from the spatio - temporal dimension.

**Figure 3:**
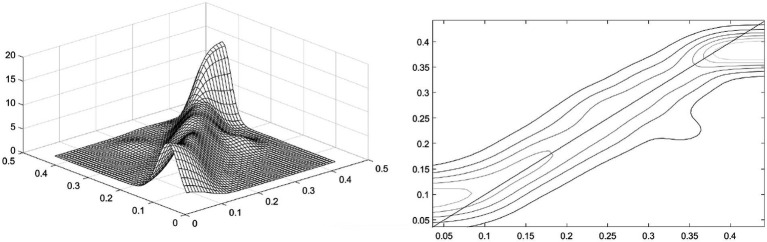
Spatial static Kernel density and its contour lines.

Spatial static Kernel density estimation is used to investigate the mutual influence of the development of health services among regions under the condition of spatial lag. Figure 3 is the spatial static Kernel density and its density contour map, respectively. The X-axis represents the development level of health services in geographically adjacent regions, and the Y-axis represents the development level of health services in the local region. As shown in Figure 3, overall, the transition probabilities of the health service level are concentrated on both sides of the 45-degree diagonal line. However, it is worth noting that within the intervals of low and high health-service levels of adjacent regions, some transition probabilities exhibit a characteristic parallel to the X-axis. This indicates that, overall, the development levels of health services in adjacent provincial regions show similarity. There is a certain spatial spillover effect as neighboring provincial regions influence each other. While in the low-value and high-value intervals of the health service level, although adjacent regions show a certain degree of similarity, the mutual influence between regions is significantly weakened.

**Figure 4:**
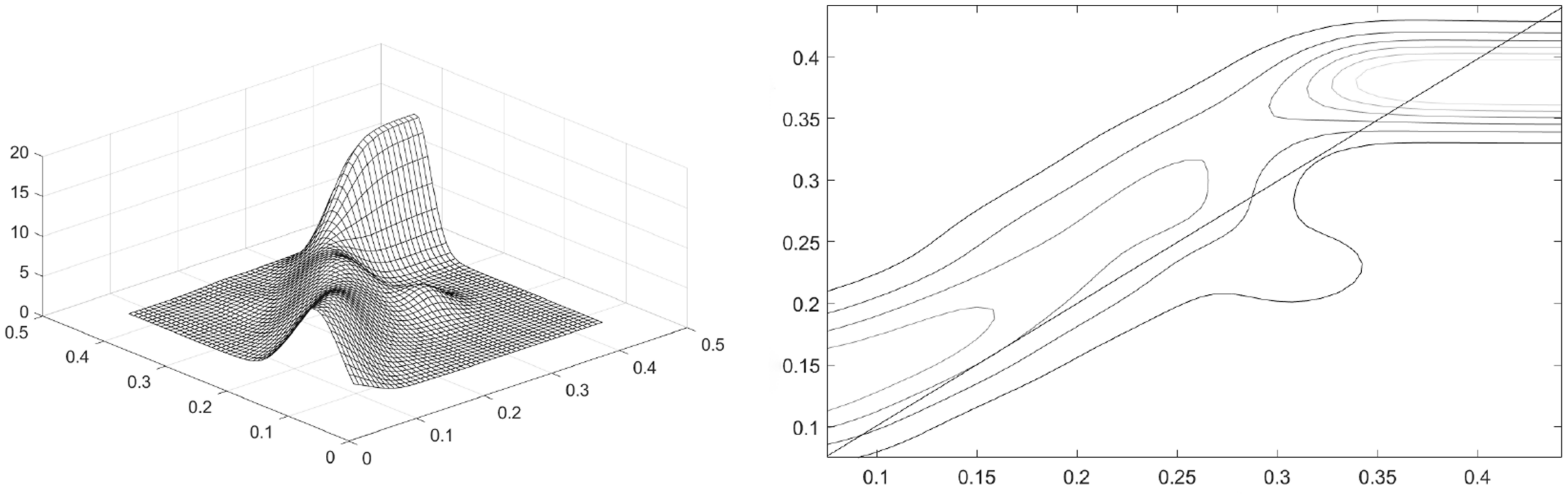
Spatial dynamic Kernel density and its contour lines.

Spatial dynamic Kernel density estimation is employed to explore the impact of the development level of health services in adjacent regions on the development level of health services in the local region 3 years later (*t* + 3) under the condition of spatial lag. Figure 4 is, respectively, the spatial dynamic Kernel density and contour map of the development level of China’s health services. The X-axis represents the development level of health services in adjacent regions at time t, and the Y-axis represents the development level of health services in the local region at time *t* + 3. Overall, the dynamically estimated Kernel density and its contour lines under spatial conditions are basically consistent with the results of the static Kernel density estimation under spatial conditions. This indicates that the spatial interaction of the development of health services in different provincial regions of China is continuous. The development of health services in adjacent provincial regions at time *t* is not only related to the development of health services in the local provincial region at time *t*, but also shows a significant spatial spillover effect with a 3-year lag.

### 4.2 Spatial decomposition of development differences

In this section, the Theil index is used to measure the spatial differences in the development of China’s health services, and the sources of these spatial differences are decomposed. Tables 2, 3 present the calculation results of the Theil index and contribution rate of the development of health services across the whole country and the three major regions.

**Table 3:** The Theil index and its decomposition.

Year	Theil index	Intra-regional	Inter-regional	East	Central	West
2005	0.0356	0.0251	0.0105	0.0330	0.0165	0.0196
2006	0.0314	0.0208	0.0106	0.0274	0.0149	0.0154
2007	0.0280	0.0194	0.0086	0.0276	0.0141	0.0117
2008	0.0255	0.0170	0.0085	0.0243	0.0112	0.0110
2009	0.0187	0.0121	0.0067	0.0158	0.0083	0.0099
2010	0.0166	0.0112	0.0054	0.0145	0.0059	0.0110
2011	0.0146	0.0097	0.0050	0.0130	0.0040	0.0097
2012	0.0127	0.0090	0.0036	0.0110	0.0052	0.0096
2013	0.0110	0.0081	0.0029	0.0110	0.0044	0.0074
2014	0.0101	0.0074	0.0027	0.0105	0.0042	0.0061
2015	0.0096	0.0071	0.0025	0.0106	0.0049	0.0047
2016	0.0095	0.0072	0.0023	0.0106	0.0051	0.0047
2017	0.0086	0.0066	0.0020	0.0105	0.0045	0.0036
2018	0.0077	0.0061	0.0016	0.0098	0.0038	0.0036
2019	0.0073	0.0061	0.0012	0.0097	0.0031	0.0044

As Table 3 clearly shows, during the period from 2005 to 2019, the Theil index of China’s health services demonstrates a significant downward trend. Specifically, its highest value reaches 0.0356 in 2005, while the lowest value is 0.0073 in 2019. Moreover, throughout the sample period, the intra-regional Theil index consistently remains higher than the inter-regional Theil index. The inter-regional Theil index exhibits a slow downward trend, while the intra-regional Theil index demonstrates a significant downward trend. The trend of the intra-regional Theil index is similar to that of the overall Theil index, and there is a substantial decline from 2008 to 2009. This indicates that the spatial differences in the development of China’s health services are significantly shrinking over time, and the differences within regions are greater than those between regions. Among the Theil indices of the three major regions, the Theil index of the eastern region is the highest and experiences the largest decline, with a significant drop from 2008 to 2009, which is consistent with the significant downward trend of the inter-regional Theil index during this period. The Theil index of the central region shows a significant downward trend before 2011 and fluctuates at a stable level after 2011. The Theil index of the western region shows a step-wise downward trend.

As can be seen from Table 4, the contribution rate of the intra-regional Theil index in the three major regions is much higher than that of the inter-regional Theil index, and the gap between the two continuously widens. The contribution rate of the intra-regional Theil index generally shows a slow upward trend. Specifically, it fluctuates significantly from 2005 to 2014, with both increases and decreases, and then steadily rises since 2014. The contribution rate of the inter-regional Theil index generally shows a slow downward trend. From 2005 to 2014, it fluctuates in the opposite direction to the contribution rate of the intra-region, and then steadily decreases since 2014. Regarding the contribution rates of the Theil index in the three major regions, the contribution rate of the eastern region far exceeds that of the central and western regions. Its average value is 42.61, and the contribution rate continuously increases since 2012. The average contribution rate of the central region is 10.99, with relatively small and stable changes. The average contribution rate of the western region is 18.30, and its trend shows greater fluctuations compared with that of the central region.

**Table 4:** Sources and contribution rates of spatial development imbalance in health services.

Year	Intra-regional	Inter-regional	East	Central	West
2005	70.40	29.60	42.68	10.47	17.24
2006	66.26	33.74	40.15	11.12	15.00
2007	69.35	30.65	44.26	11.93	13.16
2008	66.67	33.33	42.62	10.59	13.46
2009	64.40	35.60	36.68	10.95	16.77
2010	67.67	32.33	37.30	8.98	21.39
2011	66.01	33.99	37.50	6.84	21.67
2012	71.37	28.63	35.64	10.67	25.06
2013	73.66	26.34	40.58	10.15	22.93
2014	73.49	26.51	42.03	10.66	20.80
2015	74.36	25.64	44.51	12.87	16.98
2016	75.53	24.47	44.82	13.48	17.24
2017	76.54	23.46	48.78	13.15	14.61
2018	79.18	20.82	50.17	12.40	16.61
2019	83.67	16.33	51.49	10.66	21.52

In conclusion, intra-regional differences are the main source of the spatial disparities in the development of China’s health services. At the regional level, compared with the central and western regions, the eastern region has larger intra-regional differences and contributes the most to the overall disparities. In terms of trends, the spatial differences in the development of China’s health services, along with intra-regional and inter - regional differences, all exhibit a gradually decreasing trend.

### 4.3 Structural decomposition of development differences

The spatial decomposition of development differences in health services in the previous section reflects the composition of differences in geographical sense. However, it cannot reflect the source of development differences in economic sense. In fact, health services include basic categories such as personnel, facilities, curative ability, primary services and service utilization. At both the national and regional levels, the differences in development originate from these five dimensions. Therefore, in this section, from the perspective of structural decomposition and using the variance decomposition method, this paper analyzes the structural sources of development differences in China’s health services.

**Figure 5:**
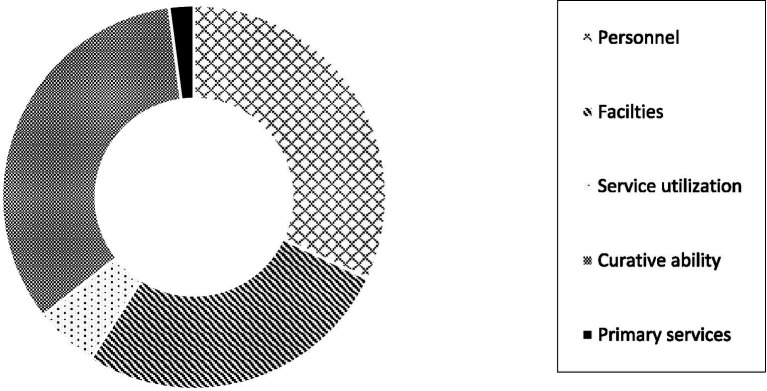
Structural decomposition at the national level.

The structural decomposition results of development differences in China’s health services are shown in Figure 5. At the national level, the primary source of development differences in China’s health services is curative ability, with an average contribution rate of 33.51%. Developed provinces possess more advanced medical technologies and professional nursing, and are able to carry out various high - difficulty surgeries and cutting-edge treatment techniques. In underdeveloped areas, however, some complex diseases may not be effectively diagnosed and treated, and patients need to be transferred to developed regions. The second source of differences is personnel, with an average contribution rate of 32.02%. The third source of imbalance is the difference in facilities, with an average contribution rate of 27.02%. The contribution degrees of service utilization and primary services are relatively small, with average contribution rates of 5.38 and 2.08%, respectively.

**Figure 6:**
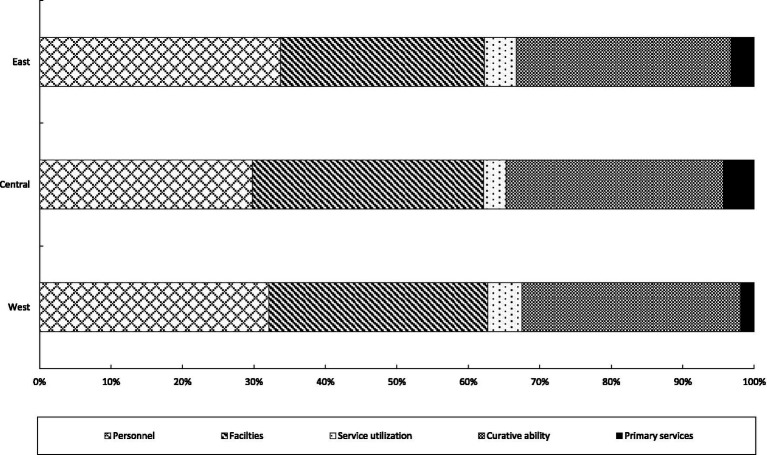
Structural decomposition at the regional level.

From the regional level, as shown in Figure 6, there are certain differences in the sources of development imbalance in the three major regions. Specifically, in the eastern region, the main source of development differences in health services is personnel, with an average contribution rate of 33.72%. The second source is curative ability, with an average contribution rate of 30.05%. In the central region, the primary source of development differences in health services is facilities, with an average contribution rate of 32.33%. The secondary source is curative ability, with an average contribution rate of 30.38%. In the western region, the primary source of differences is personnel, with an average contribution rate of 32.09%. It is followed by facilities and curative ability, with average contribution rates of 30.63 and 30.60%, respectively. It can be seen that whether at the national level or within the three major regions, personnel, facilities and curative ability are all important sources of development differences in health services, while the influence of service utilization and primary services is relatively small. The above results indicate that the allocation and services of personnel and facilities are the keys to promoting the equalization of health services in China.

## 5 Formation mechanism for differences in the development of health services in China

The previous analysis results indicate that there are significant differences in the development of health services in China, but the specific reasons for these differences have not yet been answered. In this section, the QAP regression analysis is utilized to disclose the driving impacts of differences in personnel, facilities, service utilization, curative ability, and primary services on the disparities in the development of China’s health services.

The results of QAP regression analysis based on 5,000 random permutations are shown in Table 5. All five factors passed the 1% significance level test, indicating that differences in any field between provinces will lead to differences in the development of health services, and the expansion of differences in any factor will promote the expansion of differences in health development. Specifically, the expansion of differences in personnel, facilities, service utilization, and curative abilities will to varying degrees widen the disparities in the development. In contrast, narrowing the gap in the development of primary services cannot effectively promote the equalization of health services in China.

**Table 5:** The results of QAP regression analysis.

Variables	Nation wide		Eastern region		Central region		West region	
	Coefficient	*p*-value	Coefficient	*p*-value	Coefficient	*p*-value	Coefficient	*p*-value
Primary services	−0.025	0.001	0.053	0.001	−0.141	0.000	0.041	0.001
Facilities	0.186	0.000	0.492	0.000	0.348	0.000	0.228	0.000
Service utilization	0.083	0.000	0.083	0.000	0.003	0.348	0.096	0.000
Personnel	0.600	0.000	0.256	0.000	0.491	0.000	0.591	0.000
Curative ability	0.413	0.000	0.369	0.000	0.398	0.000	0.355	0.000

In terms of intensity, the dimension of personnel has the greatest impact on the imbalance of the development level of health services, with a standardized regression coefficient of 0.600. Therefore, targeted enrollment and inter-school cooperation can be carried out in underdeveloped areas through the support of institutions of higher learning, and on-the-job training can be strengthened. In terms of talent introduction and incentives, preferential policies can be introduced to attract outstanding talents and give full play to the residual heat of retirees. In addition, through the construction of telemedicine and informatization, a telemedicine platform can be built to strengthen the application of informatization and the sharing of online resources. It is also possible to promote regional cooperation, establish pairing assistance relationships, encourage health staff to participate in academic exchange activities, and support the holding of academic conferences locally. Subsequently, the standardized regression coefficients of curative ability and facilities are 0.413 and 0.186, respectively. In contrast, service utilization and primary services have relatively small influences, with standardized regression coefficients of 0.083 and − 0.025, respectively. Thus, it can be seen that the difference in personnel is the primary factor causing the imbalance in the development of health services in China. Therefore, balancing the allocation of health personnel in various regions can promote the equalized development of health services in various regions of China to the greatest extent. Moreover, the difference in curative ability is also an important factor causing the development disparity of health services in China. Reducing the difference in curative ability in various regions is also an effective way to promote the equalized development of health services.

This article further analyzes the impacts of the five dimensions at the regional level. The results are as shown in Table 5. In the eastern region, the differences in all five dimensions have a positive impact on the overall difference. Specifically, the difference in facilities has the greatest impact, with a standardized regression coefficient of 0.492. Followed by the differences in curative ability and personnel, the standardized regression coefficients are 0.369 and 0.256, respectively. In contrast, the effects of service utilization and primary services are relatively small, with standardized regressions of 0.083 and 0.053, respectively. Therefore, the top priority in the eastern region is to balance the allocation of health facility resources and at the same time pay attention to promoting the equalization of curative ability and health personnel. In the central region, the difference in personnel has the greatest impact, with a standardized regression coefficient of 0.491. The impacts of curative ability and facilities are relatively small, with standardized regression coefficients of 0.398 and 0.348, respectively. The impact of differences in service utilization is extremely minimal, with a regression coefficient of only 0.003. Therefore, the top priority for promoting the equalization of the development level of health services in the central region is to balance the allocation of health personnel. At the same time, attention should be paid to further promoting the equalization of curative ability and facilities. In the western region, the differences in all five dimensions have a positive impact on the imbalance of the development of health services. Specifically, the difference in personnel has the greatest impact, with a standardized regression coefficient of 0.591. Followed by the differences in curative ability and health facilities, their standardized regression coefficients are 0.355 and 0.228, respectively. The impacts of differences in service utilization and primary services are relatively small, with standardized regression coefficients of 0.096 and 0.001, respectively. Therefore, consistent with the central region, the western region should focus on the equalization work in the dimensions of personnel, curative ability, and facilities. In addition, compared with the central region, the western region should pay more attention to the problem of imbalance in the allocation of health personnel.

## 6 Conclusion

Against the backdrop of unbalanced regional development in China, it is extremely urgent to promote the balanced development of China’s health services to ensure equitable access to health for the people. This paper constructs a comprehensive evaluation index system for the development of China’s health services. The two-stage entropy method is applied to measure the development level of health services in each province of China. Subsequently, the Theil index and its decomposition method are used to examine the sources of differences in the development of China’s health services from a spatial perspective, and the variance decomposition method is employed to explore the sources of differences in the development of health services from a structural perspective. Finally, the QAP is utilized to reveal the formation mechanism behind the differences in the development of China’s health services. The main research conclusions are as follows:

First, regarding the development trend, during the sample period, the development levels of health services for the country as a whole and in the three major regions of the east, central, and west all show a distinct upward trend. Nevertheless, there are significant disparities among the three regions. The eastern region consistently has the highest level of health service development, and the western region always has the lowest. In terms of distribution, there is a trend of decentralization, presenting a scenario of “prospering in multiple locations.”

Second, from the perspective of spatial decomposition, during the study period, the Theil indices of the whole country and the three major regions (the east, central, and west) all show a distinct downward trend, indicating that the issue of spatial disparities in the development of China’s health services has improved. In terms of the source of spatial differences, the intra-regional differences are the main source of the overall disparities in the development of China’s health services. Compared with the central and western regions, the eastern region has relatively larger internal differences, contributing the highest proportion to the overall disparities.

Third, from the perspective of structural decomposition, the difference in curative ability is the main source of the disparities in the development of China’s health services. Next is the difference in personnel, with the contribution levels of the two being not significantly different. The contributions of differences in service utilization and primary services are relatively small. At the regional level, the eastern region needs to pay attention to personnel and curative ability. The central region should focus on facilities and curative ability. As for the western region, it is necessary to give priority to the disparities in personnel, facilities, and curative ability.

Fourth, regarding formation mechanism, the driving forces of differences in personnel, curative ability, facilities, service utilization, and primary services on the imbalance in the development of health services decrease in turn. From a regional perspective, in the eastern region, the dimension of facilities is the most important driving factor for the overall imbalance in the development of health services, followed by differences in curative ability. In the central and western regions, differences in personnel have the greatest impact on the overall difference in the development of health services, followed by differences in curative ability.

As a developing country with a large population, China has prominent problems of unbalanced regional development. This paper takes the development differences in China’s health services as the research object. The research conducted not only helps to promote health equity in China, but also has reference significance for the balanced development of health services globally, especially in developing countries. This paper attempts to put forward the following suggestions: First, based on the objective fact that the development level of health services in the economically developed eastern regions is significantly higher than that in the central and western regions, reasonable means such as fiscal transfer payments and resource-sharing should be adopted to assist the central and western regions in improving their health service levels. Second, based on the objective fact that the differences in health personnel are the most important source contributing to the overall differences, health cooperation among regions should be further promoted to facilitate the experience-sharing, exchange and learning of health personnel among regions. Third, at the intra-regional level, the “one-size-fits-all” governance model should be avoided, and personalized action plans should be formulated according to the non-equilibrium characteristics of health service development in different regions. For example, the eastern and central regions should focus on solving the problem of non-equilibrium development of health facilities and personnel within the regions, while the western region should focus on promoting the equalization of personnel allocation.

There are still some areas that can be further explored in this study. First, the COVID-19 pandemic has an impact on the health system. The development levels of health services in different regions may be closely related to the severity of the pandemic outbreak, which could lead to new changes in the trend of health equalization. Second, this study has examined the internal driving forces behind the differences in health services. It is necessary to conduct further analysis of external driving forces such as economic development, industrial structure, and population size. Third, this study has analyzed the differences and driving forces of the development of health services from a static perspective. It is necessary to conduct further investigations from a dynamic perspective. Fourth, machine learning methods such as random forest can be applied in future research to more accurately identify the influencing factors of the development differences in health services and predict the equalization trend of health service development.

## Data Availability

The raw data supporting the conclusions of this article will be made available by the authors, without undue reservation.
